# Rhein restores the sensitivity of *mcr-1* carrying multidrug-resistant *Escherichia coli* to colistin

**DOI:** 10.3389/fmicb.2025.1586553

**Published:** 2025-06-16

**Authors:** Nannan Wang, Dan Yang, Lijuan Cao, Xinghong Zhao, Xu Song, Xun Zhou, Renyong Jia, Yuanfeng Zou, Lixia Li, Cheng Lv, Bo Jing, Zhongqiong Yin

**Affiliations:** ^1^Department of Pharmacy, Natural Medicine Research Center, Sichuan Agricultural University, Chengdu, China; ^2^Key Laboratory of Animal Disease and Human Health of Sichuan Province, Sichuan Agricultural University, Chengdu, China

**Keywords:** rhein, colistin, *mcr-1*, antibiotic adjuvant, antimicrobial resistance, therapeutic effect

## Abstract

The emergence of the colistin resistance gene *mcr-1* has resulted in a significant reduction in the clinical efficacy of colistin against infections caused by multidrug-resistant Gram-negative pathogens. A cost-effective approach for restoring the efficacy of antibiotics is to formulate synergistic antibiotic combinations with natural compounds that target the resistance of multidrug-resistant bacteria. In this study, we have demonstrated that rhein can effectively restore the sensitivity of *mcr-1*-positive *Escherichia coli* to colistin, both *in vitro* and *in vivo*. Mechanism studies have demonstrated that rhein primarily damages bacterial cell membranes, disrupts proton motive force, and generates excessive reactive oxygen species, and down-regulates the *mcr-1* gene in *E. coli*. Compared to monotherapy, the combination of rhein and colistin greatly increased the survival rate of *E. coli* infected mice and significantly reduced the bacterial load in the viscera of the mice. Our results confirm that rhein serves as a promising adjuvant to colistin and, in combination with colistin, provides a viable approach to combat infections caused by colistin resistant *E. coli*.

## 1 Introduction

Antimicrobial resistance represents one of the most pressing challenges to global health in the 21st century (Carfrae et al., 2023). Polymyxins are currently the last-line therapy for severe infections caused by gram-negative bacteria (Materon and Palzkill, 2023). Cationic cyclic polypeptides demonstrate strong antibacterial effects against various microbial pathogens, with polymyxin E and polymyxin B serving as principal clinical agents. The lipid A component of lipopolysaccharide (LPS) in gram-negative organisms becomes the target of polymyxin E binding through electrostatic interactions (Liu et al., 2016). However, the presence of phosphoethanolamine transferase, encoded by the colistin resistance gene (*mcr-1*), reduces the negative charge of lipid A, leading to acquired resistance to colistin (Liu et al., 2016). Following the discovery of *mcr-1*, other *mcr* variants subsequent (*mcr-2* to *mcr-10*) have emerged (Sun et al., 2018). The worldwide dissemination of these resistance determinants, with a notable prevalence rate of 45% in China, critically undermines colistin therapeutic outcomes, resulting in treatment failures for infections caused by carbapenem-producing Enterobacterales (Falgenhauer et al., 2016; Sun et al., 2018). The discovery of novel antibiotics is constrained by financial costs and long development times, highlighting the need for alternative strategies such as identifying adjuvants that can restore the efficacy of existing antibiotics and improve clinical outcomes for patients with antibiotic-resistant infections (Liu et al., 2019; Tyers and Wright, 2019). Therefore, the development of effective colistin adjuvants has become a critical priority in the fight against colistin resistance.

Rhein, a natural compound derived from roots and rhizomes of Rhubarb, exhibits a diverse range of biological activities (Zhuang et al., 2020). Rhein has been found to modulate host inflammatory responses through inhibition of cytokine production, particularly of pro-inflammatory molecules including TNF-α, IL-1β, and IL-6 (Zheng et al., 2019). Notably, the bioactive compound exhibits dual immunomodulatory functions by downregulating transcriptional activity of proinflammatory cytokine genes while concomitantly upregulating concentrations of immunoregulatory factors including IL-10 (Mu et al., 2021). Additionally, rhein exhibits antibacterial activity against Gram-positive bacteria, while its structural analogs—such as a pyridinium quaternary ammonium salt derivative of rhein—have demonstrated bactericidal effects against certain Gram-negative bacteria (Yin et al., 2022). According to the literature, the combined use of rhein and ampicillin or cloxacillin shows significant synergistic antimicrobial effects against methicillin-resistant *Staphylococcus aureus* (MRSA). Additionally, rhein combined with metronidazole demonstrates a synergistic effect against *Porphyromonas gingivalis* (Azelmat et al., 2015; Joung et al., 2012). However, its efficacy against *E. coli* remains limited, and there is limited research on its role as an adjuvant when combined with antibiotics. Consequently, investigating the potential of rhein as a colistin adjuvant for treating colistin-resistant bacterial infections is of considerable importance.

A cost-effective strategy to restore antibiotic efficacy is the identification of antimicrobial enhancers for existing antibiotics. Rhein has recently garnered significant attention due to its potent anti-inflammatory properties and its ability to exhibit synergistic antimicrobial effects when combined with antibiotics such as ampicillin. However, research on the synergistic effects of rhein has predominantly focused on Gram-positive bacteria, and studies investigating the combination of rhein with antibiotics in *E. coli* are limited. Preliminary studies in our laboratory have demonstrated that rhein exhibits a synergistic effect with colistin. Therefore, this study further evaluates the efficacy of rhein in combination with colistin for treating *mcr-1* positive *E. coli* infections. Additionally, we investigated the potential mechanisms driving this synergistic effect. Our results further confirm the safety of this combination and highlight the *in vivo* therapeutic outcomes when rhein is used as an adjunct to colistin therapy.

## 2 Materials and methods

### 2.1 Bacteria strains

*E. coli* B2 (harboring the *mcr-1* gene) and *E. coli* 16QD (also carrying the *mcr-1* gene) were provided by China Agricultural University (Song et al., 2020). *E. coli* CVCC 3749 was purchased from the China General Microbiological Culture Collection Center. Rhein (with a minimum purity of 98%) was sourced from Chengdu Herbpurify Co., Ltd., located in Chengdu, China. All bacterial strains were subsequently cultured in Mueller-Hinton Broth (MHB) or on Luria-Bertani (LB) agar plates for further experimentation.

### 2.2 Reagents

Colistin, ONPG, and 3,3’-Dipropylthiadicarbocyanine iodide [DiSC_3_(5)], were sourced from Beijing Solarbio Science & Technology Co., Ltd. located in Beijing, China. ROS kit, ATP kit and cell counting kit-8 were sourced from Beyotime Biotechnology Co., Ltd. in Shanghai, China. Furthermore, ELISA kits were obtained from Shanghai-based mlbio Biotechnology Co., Ltd.

### 2.3 Checkerboard assays

To determine the synergistic interactions between colistin and rhein, a modified checkerboard method was employed as previously described (Huang et al., 2023; Odds, 2003). The concentration range for both agents was adjusted within 1/32 MIC to 4 MIC. Each well of a 96-well microplate was filled with 100 μL of sterilized MHB. Rhein were introduced into the final row and subsequently diluted to the second row. Similarly, colistin were administered to the first column and diluted across to the seventh column. Subsequently, each well received an inoculum containing 10^6^ CFUs of the bacterial suspension in 100 μL volume. Following incubation for 18 h, optical density measurements were obtained at 600 nm employing a microplate reader. The FIC index (FICI) was computed through the equation: FICI = FIC_a_ + FIC_b_ = MIC_a_ in combination / MIC_a_ alone + MIC_b_ in combination/MIC_b_ alone. Interpretation criteria were established as follows: synergy (FICI ≤ 0.5), indifference (0.5 < FICI ≤ 4), and antagonism (FICI > 4). This standardized protocol was implemented to assess colistin-rhein combinations against *E. coli*, with four independent experimental replicates conducted.

### 2.4 Time-kill curve

*E. coli* B2 was cultured overnight, then diluted with MHB to a bacterial solution concentration of 1 × 10^6^ CFU/mL and incubated at 37°C. Following the combination effect analysis, the bacterial cultures were subjected to different concentrations of rhein (40 and 320 μg/mL), colistin (0.5 and 1 μg/mL), or a combination of these drugs (rhein/colistin: 40 and 0.5 μg/mL, 40 and 1 μg/mL, 40 and 2 μg/mL, 320 and 1 μg/mL) for 24 h. At predetermined intervals (0, 2, 4, 6, 8, 12, 24 h), 100 μL aliquots were collected, centrifuged, and then resuspended in 10 mM phosphate-buffered saline (pH 7.4). This suspension was subjected to 10-fold serial dilutions and transferred onto LB agar plates for incubation at 37°C overnight. The bacterial colonies were counted to generate a time-killing curve. The combined treatment was deemed synergistic if it demonstrated ≥2 log_10_ CFU/mL higher killing efficiency compared to the most effective single agent. If the combined effect resulted in ≥2 log_10_ CFU/mL less killing than the best single drug, it was classified as antagonistic. When the combined effect varied by ≤2 log_10_ CFU/mL relative to the individual agents, the interaction was considered indifferent (Cuenca-Estrella, 2004; Vitale et al., 2005).

### 2.5 Biofilm inhibition and eradication assay

To evaluate biofilm inhibition, *E. coli* B2 in the exponential growth phase was standardized to an optical density (OD_600_) of 0.5. After standardization, the bacterial suspension was subjected to a 1: 100 dilution in LB broth. Aliquots of diluted specimens were allocated to 96-well microtiter plates preloaded with antimicrobial agents at specified concentrations: colistin (0.5 and 1 μg/mL), rhein (40 and 320 μg/mL), or combinatorial preparations (rhein + colistin: 40 + 0.5 and 320 + 1 μg/mL), and incubated for 24 h at 37°C. After incubation, the plates were gently washed with PBS, air-dried, and stained with 0.1% crystal violet. The bound dye was solubilized using 30% acetic acid, and the absorbance at OD_595_ was measured to quantify biofilm formation.

Biofilm eradication assay: *E. coli* B2 was cultured overnight in 96-well plates for 24 h to allow biofilm formation. Mature biofilms underwent dual PBS washing cycles before treated with rhein (40/320 μg/mL), colistin (0.5/1 μg/mL), or combination (10 + 0.5 or 320 + 1 μg/mL for rhein and colistin respectively), followed by an additional 24 h of incubation at 37°C. After incubation, each well was rinsed twice with 1 × PBS, and a crystal violet assay was performed to quantify the viable cells remaining in the biofilm. Biofilm eradication was calculated by comparing the OD_595_ values of antibiotic-treated biofilms to those of untreated controls (Haney et al., 2021).

### 2.6 Scanning electron microscopy

Samples of *E. coli* B2 were subjected to exposure under three experimental conditions: colistin (1 μg/mL, 1/4 MIC), rhein (320 μg/mL, 1/4 MIC), and their combination, with an incubation period of 4 h. Subsequently, the bacterial samples were fixed at 4°C for 24 h utilizing 2.5% glutaraldehyde. A series of sequential ethanol dehydration steps at increasing concentrations (30, 50, 70, 90, and 100%) were then performed. To achieve optimal sample preparation, critical point drying was applied followed by surface modification through gold–palladium sputter coating, enabling subsequent microscopic examination using a SEM (JSM-IT700HR).

### 2.7 Resistance development studies

*E. coli* B2 cultures in logarithmic phase were resuspended in fresh MH broth at a 1:1000 ratio. The suspensions were supplemented with subinhibitory concentrations (1/4 MIC) of either colistin alone (320 μg/mL) or combined colistin-rhein. Antimicrobial susceptibility testing was performed through broth microdilution in 96-well plates following 24 h incubation at 37°C. Subsequent passages involved transferring cultures to media containing adjusted 1/4 MIC drug concentrations. This method was applied consistently over a 30 days period, during which the change in the MIC of colistin compared to its initial MIC was assessed. All experiments were conducted with biological replicates.

### 2.8 MCR-1 expression assay

*E. coli* B2 was grown to the early log phase and subsequently subjected to sub-MIC concentrations (1/8–1/2) of rhein for 4 h. RNA purification was conducted using phenol-chloroform reagent (Trizol) followed by reverse transcription into cDNA with ExonScript RT SuperMix. The RNA samples were normalized to the same concentration during the DNA removal step prior to cDNA synthesis. qRT-PCR was performed using UltraStart SYBR Green qPCR Mix. Gene expression levels were comparatively analyzed through the 2^−ΔΔCT^ method, employing 16S rRNA as the control.

### 2.9 Membrane permeability

The ability of ONPG to assess membrane integrity was utilized (Hao et al., 2009). A suspension of *E. coli* B2 was adjusted to an absorbance of OD_600_ = 0.5. An aliquot of 100 μL was treated with varying concentrations of rhein (40 and 320 μg/mL) and colistin (0.5 and 1 μg/mL) individually and in combination, followed by incubation at 37°C for 1 h. After this period, 100 μL of the supernatant was transferred to a 96-well microplate. A preheated cuvette containing 3 mM ONPG was then used for analysis. The o-nitrophenol concentration was determined via its absorption at 420 nm following a 30-min incubation at 37°C, using a microplate reader to quantify the degree of membrane disruption.

### 2.10 Adenosine triphosphate determination

The adenosine triphosphate (ATP) levels within *E. coli* B2 cells were assessed utilizing an optimized ATP quantification system. The bacterial cells were initially cleaned with PBS at a neutral pH (7.4). Subsequently, the cell suspension was adjusted to an OD_600_ value of 0.5. The cells were then exposed to various concentrations of the colistin (either 0.5 or 1 μg/mL alone or in combination with the rhein at concentrations of 40 or 320 μg/mL for 1 h). Following this treatment, the bacterial samples were sedimented via centrifugation, and the resulting supernatants were isolated. The detection reagents were introduced into a 96-well microtiter plate, and the mixture was permitted to incubate at room temperature for 5 min. The luminescence intensity of the supernatant was subsequently measured using an automated detection system (Multi-Mode Microplate Reader).

### 2.11 Efflux pump assay

To evaluate the impact of rhein on efflux pump activity, an ethidium bromide (EtBr) efflux assay was performed according to established protocols (Song et al., 2024). Briefly, the cells were treated with varying concentrations of rhein (40 and 320 μg/mL) and colistin (0.5 and 1 μg/mL) individually and in combination, followed by incubation at 37°C for 30 min. Then, cells were collected by centrifugation (5,000 g, 10 min, 4°C) and resuspend in sterile MHB. EB was then added to the system to a final concentration of 4 μg/mL. After standing for 5 min, fluorescence intensity tracking was implemented for 60 min with spectral parameters configured at 530 nm excitation and 600 nm emission to quantify EtBr retention dynamics.

### 2.12 Total ROS measurement

The levels of ROS were assessed using an Enhanced ATP Assay Kit. Briefly, the bacterial suspension was incubated with DCFH-DA (2′,7′-dichlorodihydrofluorescein diacetate) at 37°C for 30 min, with subsequent rinsing three times in PBS. Treated cells (190 μL) incubated with combined with 10 μL of rhein (40 and 320 μg/mL), colistin (0.5 and 1 μg/mL) individually and combined, respectively. Fluorescence was measured after 30 min using a multi-mode microplate reader, which was configured for excitation at 488 nm and emission at 525 nm.

### 2.13 Membrane depolarization assay

The temporal variations in *E. coli* B2 membrane potential were assessed using DiSC_3_(5) fluorescence (0.5 μM). Initially, bacterial cultures were diluted to an optical density of OD_600_ ≈ 0.5 via three sequential washes with PBS. Following this, the cell suspension was treated with DiSC_3_(5) under standard conditions of 37°C for a 15-min incubation period. Subsequently, the treated cells were transferred into a 96-well plate, where each well received 190 μL of bacterial suspension were mixed with 10 μL of rhein (40 and 320 μg/mL), colistin (0.5 and 1 μg/mL) or a combination of both. The fluorescence intensity was continuously monitored every 5 min, utilizing 622 nm excitation and 670 nm emission wavelengths.

### 2.14 Transcriptomic analysis

The total RNA was isolated utilizing total RNA extraction kit and subsequently sequenced using paired-end methodology on an Illumina NovaSeqXPlus platform, achieving a read length of 2 × 150 base pairs. This process enabled comprehensive analysis of the transcriptional profile of *E. coli* B2 under colistin monotherapy (1 μg/mL) and in combination with rhein (320 μg/mL) over a 5-h exposure period. The raw sequencing data underwent quality filtering and reference-based alignment against the *E. coli* B2 genome (CP082327.1). Differential expression analysis was executed via the Cuffdiff algorithm, employing normalized transcript abundance (FPKM) metrics. Genes were identified as differentially expressed when exhibiting a log_2_ fold change exceeding 1 (corresponding to >2-fold) and maintaining a statistical significance of *p* < 0.05. Experimental accuracy was ensured through biological triplicates for each treatment condition. Sequencing data has been deposited in the NCBI Sequence Read Archive under the accession number PRJNA1164984.

### 2.15 Mouse peritonitis infection model

All animal experiments conformed to the Guide for the Care and Use of Laboratory Animals from the National Institutes of Health, and all procedures were approved by the Animal Research Committee of Sichuan Agricultural University.

ICR mice aged 6 weeks (*n* = 10 per group) were inoculated intraperitoneally with a suspension of *E. coli* B2 (3.0 × 10^8^ CFUs). After 1 h, the mice were administered either PBS (pH 7.4, 10 mM), solvent (0.1% NaHCO_3_), colistin (10 mg/kg), rhein (10 mg/kg), or combinations of colistin and rhein (10 + 10 mg/kg) through intraperitoneal injection. The animals were monitored for survival status over a seven-day period. Upon succumbing, necropsy was performed, and the heart, lungs, liver, kidneys, and spleen were subjected to weighing, homogenization, and plating to determine the bacterial load.

### 2.16 Statistical analyses

The statistical analysis was executed using GraphPad Prism 8.0 software. Data values are presented as the mean alongside SD. Two-group comparisons were evaluated using unpaired *t*-tests, assuming normal distribution, while ANOVA was utilized for multi-group assessments. Significance was set at α = 0.05, with the following indicators: *p < 0.05, **p < 0.01, ***p < 0.001, ****p < 0.0001.

## 3 Results

### 3.1 *In vitro* synergistic activity of rhein and combination with colistin against *Escherichia coli*

**Figure 1:**
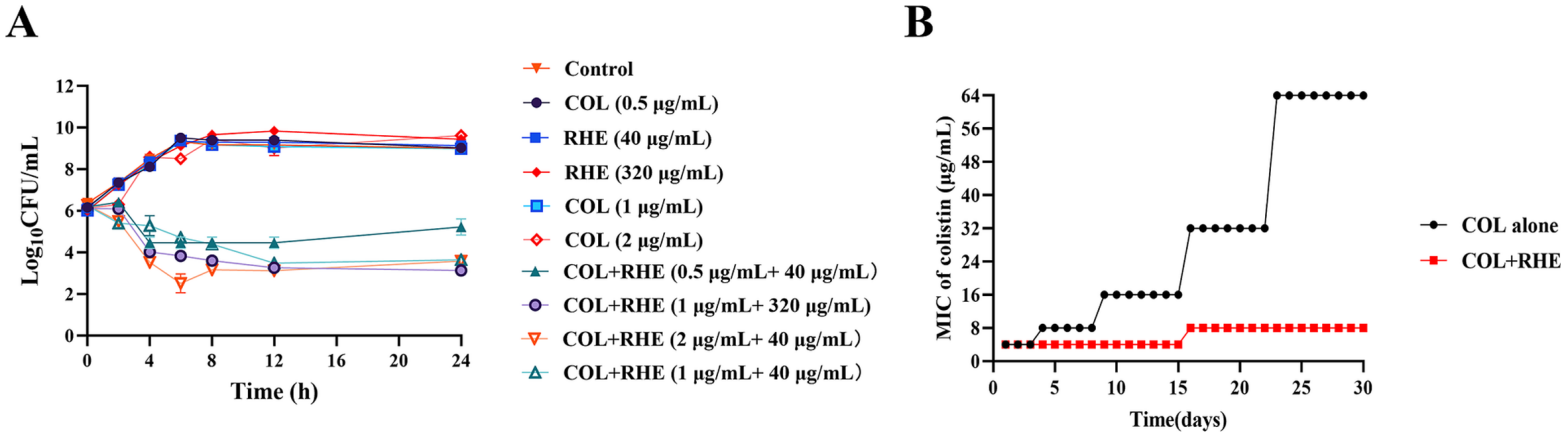
Rhein enhanced the antibacterial activity of colistin *in vitro*. **(A)** Time-killing assays of rhein and colistin against *E. coli* B2 (*mcr-1*); **(B)** MIC changing curves of *E. coli* B2 under the treatment of subinhibitory concentrations of colistin or colistin combined with rhein (320 μg/mL). COL, colistin; RHE, rhein.

To assess the synergistic effects between rhein and colistin, we performed checkerboard experiments utilizing clinical *E. coli* strains with varying colistin susceptibility profiles, including strains harboring the *mcr-1* gene. The checkerboard method showed a clear synergism between the two agents in *mcr-1*-positive *E. coli* isolates, with FIC indices ranging between 0.141 and 0.156. Notably, the combination led to a significant decrease in MIC values for colistin against *E. coli* B2 and 16QD, specifically dropping from 4 μg/mL to 0.5 μg/mL (Table 1; Supplementary Figure S1). However, no such interaction was observed when testing against colistin-sensitive *E. coli* CVCC 3749. The combination of rhein and colistin with different concentrations was used to analyze the time killing effect of exponential proliferation cultures of *E. coli* B2 treated alone or in combination. While neither rhein nor colistin demonstrated bacteriocidal activity when used separately, the combination resulted in a pronounced bacterial population reduction, achieving a 4–6 log_10_ CFU/mL decrease within 4–6 h, and a sustained inhibitory effect at 24 h (Figure 1A). However, the bacteria began to grow after 8 h of inhibition by colistin (0.5 μg/mL) and rhein (40 μg/mL) on *E. coli*. Furthermore, serial passage experiments were conducted over 30 d using sub-MIC (1/4 MIC) colistin, with and without 320 μg/mL rhein. Strikingly, no resistant mutants emerged in the co-treatment group (Figure 1B), whereas sole colistin exposure led to significant resistance development, evidenced by a 16-fold MIC increase. After 15 d of induction with rhein and colistin, the MIC of *E. coli* to colistin doubled, but no statistically significant difference was observed. These findings highlight the potential of rhein to enhance colistin efficacy against *mcr-1*-positive *E. coli* while simultaneously suppressing the emergence of resistance.

**Table 1:** Combined MIC of rhein with colistin.

Strains	The MIC of colistin (μg/mL)		The MIC of rhein (μg/mL)		FICI^a^
	Alone	Combination	Alone	Combination	
*E. coli* B2 (*mcr-1*)	4	0.5	1,280	40	0.156
*E. coli* 16QD (*mcr-1*)	4	0.5	2,560	40	0.141
*E. coli* CVCC 3749 (colistin-sensitive)	0.25	0.125	2,560	40	0.516

a FICI, FIC index (FICI); FICI ≤ 0.5 indicates synergy; 0.5 < FICI ≤ 4 indicates “no interaction.

### 3.2 Rhein and colistin combination inhibit *Escherichia coli* biofilm formation

**Figure 2:**
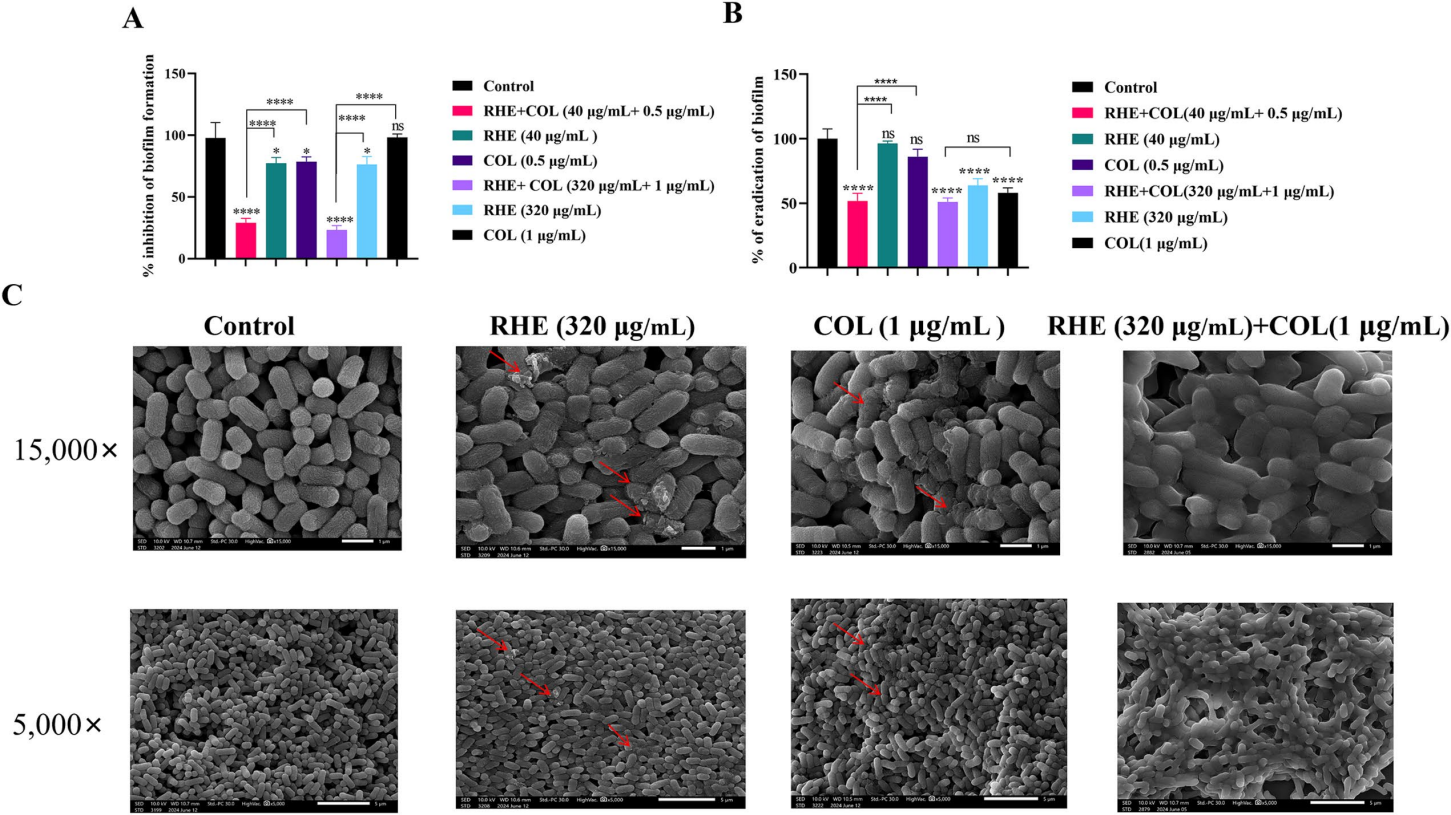
Rhein enhanced the anti-biofilm of colistin *in vitro*. **(A)** Biofilm inhibition; **(B)** Biofilm eradication; **(C)** Scanning electron microscope. *p < 0.05, **p < 0.01, ***p < 0.001, ****p < 0.0001.

The development of biofilms significantly contributes to bacterial resistance mechanisms, and rhein has been identified as a potent QS inhibitor for bacterial species (Hall and Mah, 2017). To investigate the combined effects of rhein and colistin on the formation of *E. coli* biofilm, the bacterial biofilm was observed by crystal violet staining and scanning electron microscopy (SEM). Our results demonstrated that the co-application of rhein and colistin induced a marked reduction in *E. coli* B2 biofilm development compared to either agent used individually (*p* < 0.05; Figure 2A). Furthermore, the combination exhibited enhanced biofilm eradication, with the inhibition rate of biofilm formation exceeding the clearance efficiency observed for mature biofilms (Figure 2B). To provide a more intuitive perspective on the effects of the combined treatment, we present our findings in Figure 2C. In the control group, the bacterial cells displayed their characteristic rod-shaped, smooth, and plump morphology. When treated with either rhein or colistin alone, some cells exhibited signs of structural deformation, including folding and membrane rupture. In contrast, the combined administration of rhein and colistin caused the cells to lose their original shape and aggregate, indicating significant morphological disruption. These findings collectively demonstrate that the combined application of rhein and colistin induces substantial alterations in bacterial morphology, leading to effective inhibition and eradication of biofilm formation.

### 3.3 Rhein enhanced membrane-damaging activity of colistin

**Figure 3:**
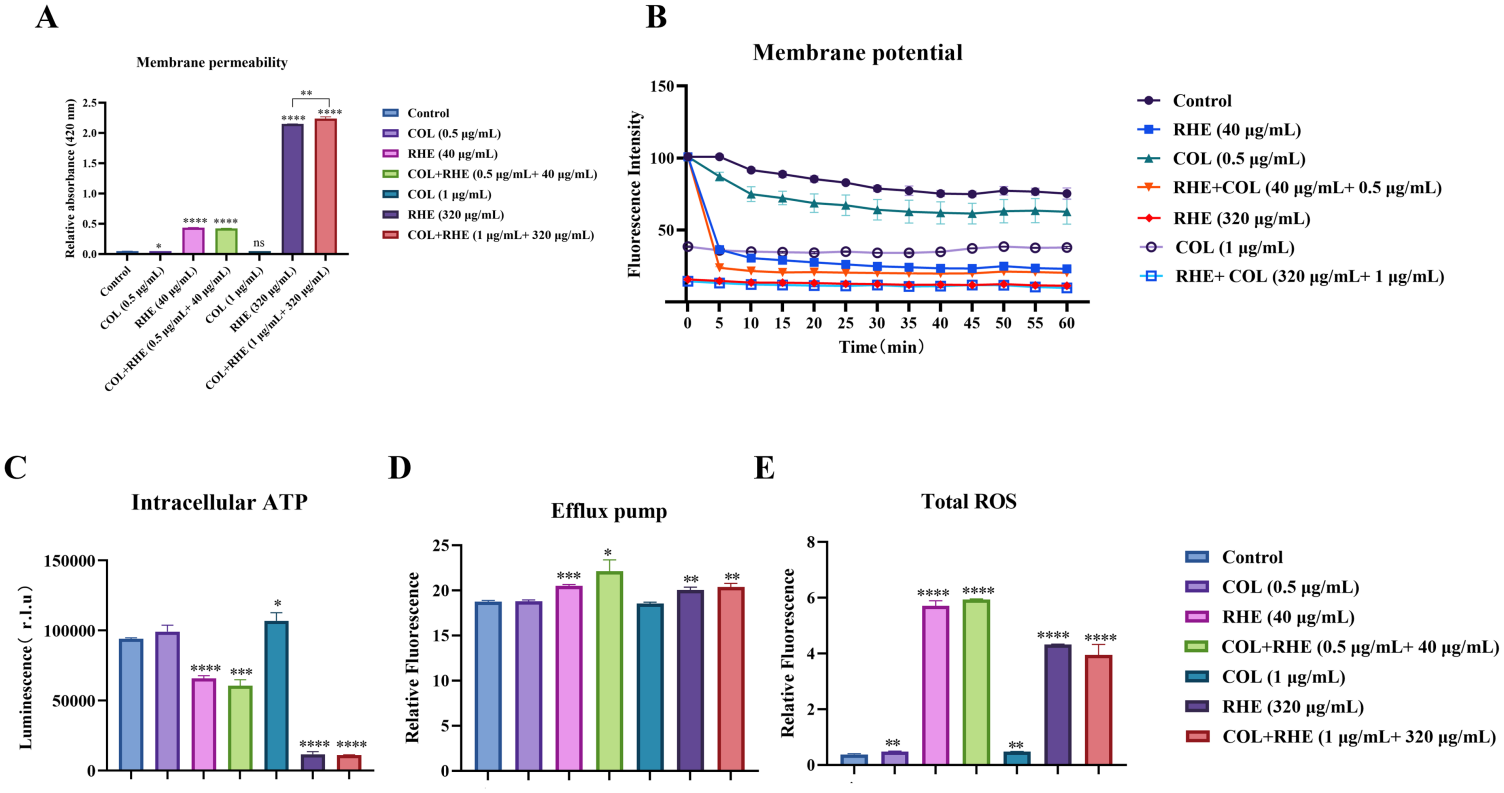
Synergistic mechanisms of colistin-rhein combination. **(A)** Permeability of membrane of *E. coli* B2 after 1 h exposure to colistin and rhein or their combination; **(B)** Membrane potential of *E. coli* B2 treated with rhein plus colistin for 1 h; **(C)** The intracellular ATP level of *E. coli* B2 after treatment with rhein plus colistin; **(D)** The efflux pump of *E. coli* B2 after treatment with rhein plus colistin; **(E)** The ROS level of *E. coli* B2 after treatment with rhein plus colistin. *p < 0.05, **p < 0.01, ***p < 0.001, ****p < 0.0001.

The resistance of bacteria to colistin is primarily attributed to alterations in lipopolysaccharides, which decrease the ability of the compounds to disrupt the bacterial outer membrane. Initially, we analyzed the impact of rhein, colistin, and their combination on bacterial membrane properties. Treatment with rhein and its combinations increased membrane permeability (Figure 3A). Subsequently, DiSC_3_(5) was utilized to assess both membrane permeability and potential. The results showed a dose-dependent decrease in plasma membrane depolarization following exposure to rhein and its combinations (Figure 3B). Accounting for the fact that the PMF is crucial for ATP generation, we observed a marked decline in intracellular ATP levels in *E. coli* after treatment with rhein (Figure 3C). Since the PMF also drives efflux pump activity, we measured pump functionality using EtBr. A modest increase in EtBr efflux was noted in *E. coli* B2 when exposed to the combination of rhein and colistin (Figure 3D). Furthermore, ROS generation was assessed as a key mechanism of bacterial mortality. Both rhein and its combinations significantly elevated ROS levels (Figure 3E). These findings imply that rhein enhances membrane permeability, disrupts PMF, and induces excessive ROS production within cells, thereby reinforcing the efficacy of colistin.

### 3.4 Inhibition of *mcr-1* gene expression and mechanism by rhein

**Figure 4:**
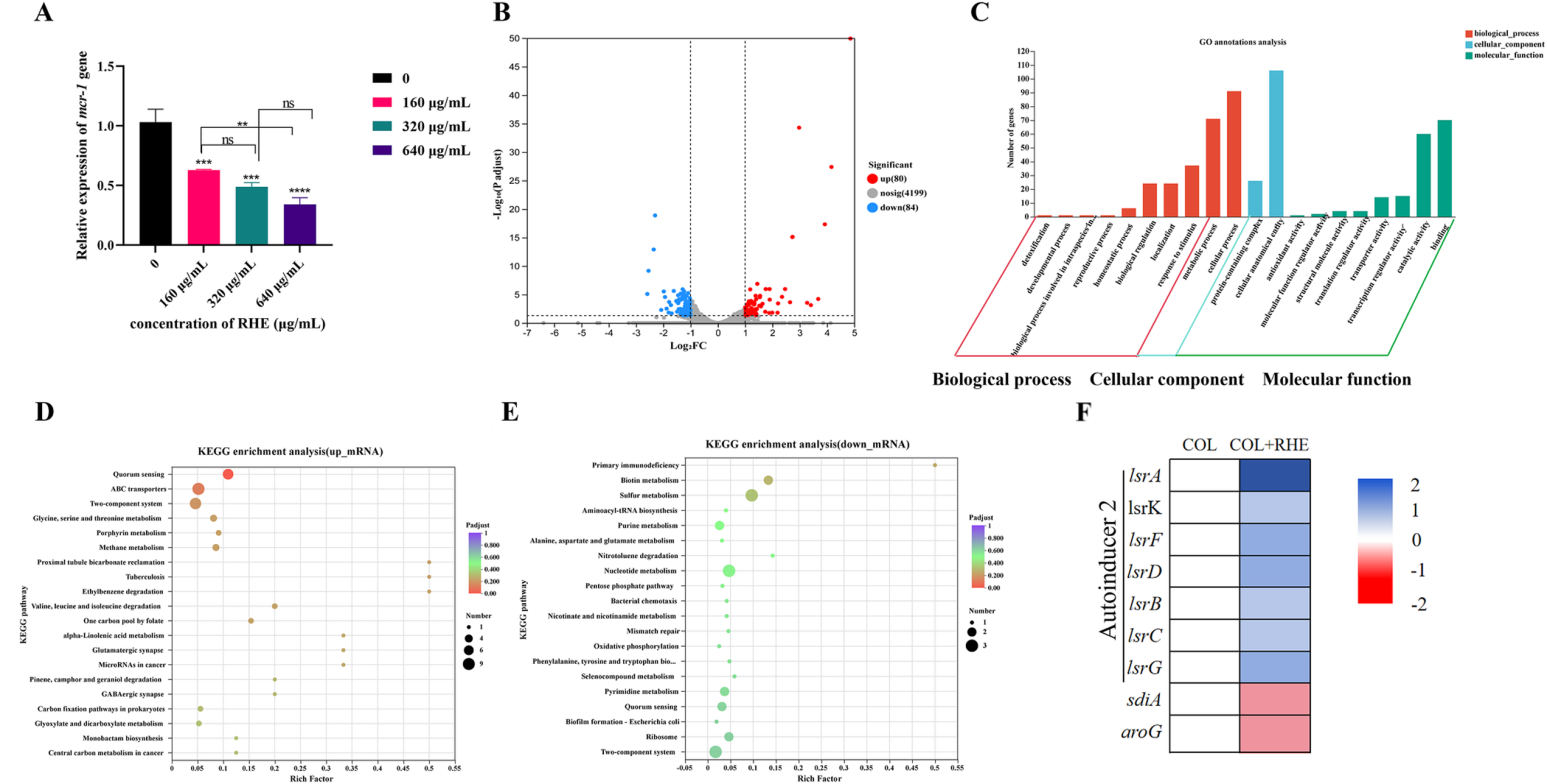
Transcriptome analysis of *E. coli* B2 after exposure to colistin alone or the combination of colistin plus rhein. **(A)** The effect of rhein treatment on the expression of *mcr-1* gene; **(B)** Volcano plot and **(C)** GO annotation analysis of the DEGs in *E. coli* B2 after exposing colistin (1 μg/mL) or the combination of colistin (1 μg/mL) plus rhein (320 μg/mL) for 4 h. KEGG enrichment analysis of **(D)** upregulated DEGs and **(E)** downregulated DEGs. The 20 most significant enriched pathways are shown. **(F)** Selected differential expression genes involved in quorum sensing. Data were presented as means of three biological replicates. COL, colistin alone; COL + RHE, the combination of colistin and rhein. *p < 0.05, **p < 0.01, ***p < 0.001, ****p < 0.0001.

The investigation of the inhibitory effects of rhein on *mcr-1* gene expression demonstrated a significant suppression of *mcr-1* transcriptional activity in *E. coli* B2, as corroborated by qRT-PCR analysis (Figure 4A). To further analyze the molecular mechanisms responsible for these transcriptional modifications, transcriptional profiling was conducted on the isolated strain after exposure to either colistin alone or a combination of colistin and rhein for 5 h. A comparative analysis was performed, contrasting the gene expression profiles of the combined treatment against colistin monotherapy, revealing 80 upregulated and 84 downregulated transcripts (Figure 4B). Gene Ontology (GO) categorization of these differentially expressed transcripts uncovered their involvement in various biological functions, including cellular and metabolic processes, as well as their association with cellular components such as cell structures, and molecular functions such as catalytic functions and binding (Figure 4C). KEGG pathway enrichment mapping indicated that the enhanced transcripts were significantly enriched in pathways linked to quorum sensing systems, ABC transporter mechanisms, and two-component regulatory networks, while the suppressed transcripts were predominantly associated with two-component signaling pathways, nucleotide biosynthesis, and sulfur metabolism (Figures 4D,E). Notably, the *lsr*-associated genes, integral to bacterial quorum sensing, were found to be transcriptionally activated under the combined colistin and rhein treatment (Figure 4F). This pattern suggests that bacteria activate specific response pathways to counteract environmental perturbations and maintain cellular stability. The findings collectively indicate that rhein as a quorum sensing inhibitor, impairing cellular membrane integrity, and altering bacterial metabolic processes, and down-regulating *mcr-1* gene expression. This dual action enhances the potency of colistin by synergistically amplifying its bactericidal effects.

### 3.5 Rhein restores therapeutic efficacy of colistin *in vivo*

**Figure 5:**
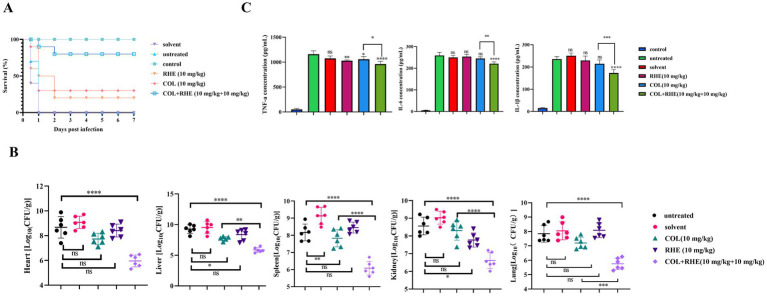
Rhein restored the therapeutic efficacy of colistin in mouse peritonitis infection model. **(A)** Survival rate of mice after monotherapy with colistin, rhein or treatment with their combination (*n* = 10). **(B)** Visceral bacterial burden of mice after treatment with colistin, rhein or their combination. COL, colistin; RHE, rhein. **(C)** The related inflammation factors were determined in blood serum after the mice were treated with colistin, rhein or their combination, including **(A)** TNF-α, **(B)** IL-6, **(C)** IL-1β. *p < 0.05, **p < 0.01, ***p < 0.001, ****p < 0.0001.

The anti-inflammatory properties of rhein and its potential as a therapeutic agent were investigated through *in vivo* efficacy testing using mouse infection models. In the peritonitis-sepsis model, mice treated with a combination of colistin and rhein (10 mg/kg) demonstrated a marked improvement in survival rates (80% at 7 days) compared to untreated controls and mice receiving colistin alone (30% survival rate; Figure 5A). This combinatorial approach also significantly reduced bacterial loads in various organ systems, with a decrease of approximately 3–4 log_10_ CFU/mL (Figure 5B). Furthermore, the expression levels of inflammatory cytokines IL-6, IL-1β, and TNF-α were measured using an ELISA kit. As shown in Figure 5C, untreated, solvent-treated, and single-drug groups exhibited elevated cytokine levels, whereas the combination therapy group displayed significantly lower cytokine expression compared to untreated controls. These results confirm that rhein significantly enhances the *in vivo* efficacy of colistin.

**Figure 6:**
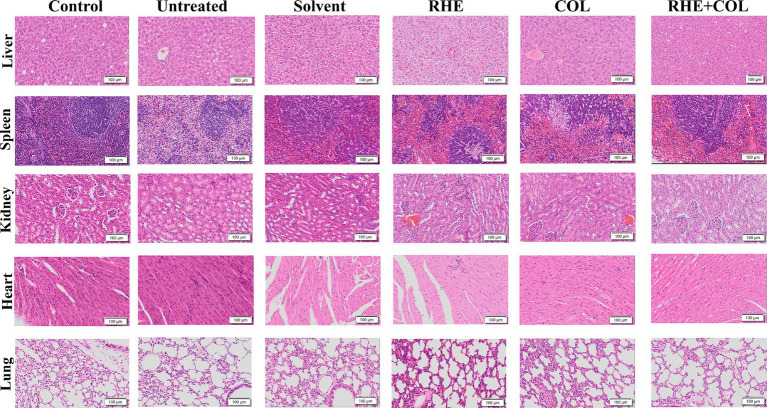
Histopathological observation of heart, liver, spleen, lung and kidney after different treatments (×200).

Histological analyses using hematoxylin and eosin (H&E) staining demonstrated that, compared to the control group, the sepsis groups (including untreated, solvent-treated, and single-drug-treated groups) exhibited histopathological changes, such as visceral organ exhibited degeneration and infiltration by inflammatory cells, confirming the successful induction of the peritonitis-sepsis model. In addition, histopathological examinations revealed signs of cell damage and vacuole formation in the liver, as well as congestion in the spleen. Additionally, all groups (control, solvent, rhein, and colistin-only groups) displayed histological features in the kidneys, including hypertrophy of renal tubular epithelial cells, hemorrhage, and the presence of inflammatory cells. Inflammatory cell infiltration was also observed in the heart and lungs. Notably, however, the combination therapy (rhein 10 mg/kg combined with colistin 10 mg/kg) resulted in no significant histopathological changes across any of the examined tissues, indicating superior therapeutic efficacy (Figure 6). These findings highlight that the combined use of rhein and colistin not only enhances the *in vivo* effectiveness of colistin but also mitigates the inflammatory response.

## 4 Discussion

The emergence of MDR bacteria has become a significant global concern, particularly in challenging the effectiveness of available antibiotics. Among these, colistin remains a cornerstone for combating infections caused by MDR gram-negative pathogens (Bastard et al., 2022). However, the emergence of MCR, a resistance gene targeting colistin, has critically hindered its clinical utility, the urgent need for new therapeutic strategies, including the restoration of the efficacy of colistin (Song et al., 2024). Concurrently, there are significant barriers to clinical use of colistin due to its pronounced nephrotoxicity and neurotoxicity in mammalian systems (Cai et al., 2023). This necessitates both the use of subtherapeutic colistin dosing and the development of colistin antidotes to maximize its therapeutic potential. As a bioactive anthraquinone present in botanical preparations including *Rheum palmatum L*, *Aloe vera*, and *Cassia angustifolia*, rhein demonstrates multimodal therapeutic effects encompassing inflammation suppression, tumor growth inhibition, fibrosis prevention, and oxidative stress mitigation (Wu et al., 2020). Notably, this compound displays microbial growth suppression across various pathogens, showing efficacy against both *Bacillus megaterium* and pathogenic strains like *Staphylococcus aureus* and *Cutibacterium acnes* (Yin et al., 2022). However, its bactericidal effect against *E. coli* remains underexplored, and its combination with colistin has not been previously investigated. In our investigation, we found that while rhein demonstrates limited standalone antibacterial properties, it enhances the efficacy of colistin against *mcr-1* gene carrying bacteria significantly, thereby reducing the required colistin dosage.

The cationic cyclic lipopeptide colistin combats bacterial infections by disturbing the structural integrity of bacterial membranes through electrostatic binding with outer membrane lipopolysaccharides (LPS). However, this interaction is reduced in bacteria where lipid A modifications are present, such as those expressing the phosphoethanolamine transferase (EptA) enzyme or the MCR enzyme, which alters lipid A phosphorylation (Ma et al., 2024). Thus, the disruption of the membrane integrity and the ensuing oxidative damage serve as critical markers of colistin antibacterial efficacy. Our experiments showed that co-administration of colistin with rhein potentiated membrane disruption and oxidative stress in *mcr-1* positive isolates more effectively than colistin alone. Rhein is capable of inhibiting multidrug efflux systems by interfering with bacterial energy generation, specifically by affecting the PMF, which is essential for ATP synthesis via the F1F0 ATPase complex and for efflux pump operation. This inhibition reduces substrate expulsion, leading to elevated intracellular concentrations of colistin in *E. coli*, enhancing its efficacy against Gram-negative pathogens. The resultant decrease in drug efflux led to a heightened intracellular concentration of colistin in *E. coli*, which is essential for the antibiotic’s effectiveness against Gram-negative bacteria, indicating that rhein holds significant promise as a colistin adjuvant.

Emerging studies suggest that metformin, a diabetic therapy, enhances the efficacy of tetracycline antibiotics—specifically doxycycline and minocycline—and demonstrates potent activity against multidrug-resistant isolates of *Staphylococcus aureus*, *Enterococcus faecalis*, *E. coli*, and *Salmonella enteritidis* (Liu et al., 2020). As a first-line oral glucose-lowering agent for type 2 diabetes, metformin notably amplifies the potency of doxycycline against various *tet*(A)-positive pathogens (Liu et al., 2020). This innovative repurposing of existing drugs presents a promising strategy to combat bacterial resistance. Our exploration identifies rhein, a derivative of diacerein, as a potential enhancer for colistin, with analogous functional benefits. Diacerein, chemically designated as 1,8-Diacetoxy-3-carboxyanthraquinone, belongs to the anthraquinone compound family and is predominantly employed in clinical practice for osteoarthritis (OA) management while exhibiting antibacterial capabilities (Fidelix et al., 2014; Fu et al., 2023). Recent findings underscore the role of diacerein in mitigating renal damage, reducing inflammation, countering oxidative stress, and inhibiting apoptosis in diabetic contexts, in addition to alleviating pain. Moreover, it shows therapeutic potential for cancer, ulcerative colitis, testicular damage, and cervical hyperkeratosis. Diacerein also functions as a synergistic component in combination therapies (Almezgagi et al., 2020). Beyond musculoskeletal applications, diacerein serves as an antibiotic enhancer. Our research results indicate a synergistic effect between rhein and colistin. In future therapeutic strategies, combining diacerein with colistin may offer a solution for *mcr-1*-positive *E. coli* drug-resistant infections. Although colistin resistance development is challenging, further prospective clinical trials are necessary to validate the *in vivo* potentiating effects of rhein in conjunction with colistin.

## 5 Conclusion

In conclusion, our study reveals that treatment with rhein successfully restores the susceptibility of *E. coli* carrying the *mcr-1* gene to colistin, as demonstrated *in vitro* and *in vivo*. Mechanistic investigations indicate rhein primarily exerts its effect by damaging bacterial cell membranes, increasing membrane permeability, disrupting PMF, inhibiting intracellular ATP synthesis, and inducing the production of ROS. Moreover, rhein interferes with the QS system in *E. coli*, down-regulating the expression of the *mcr-1* gene, which leads to the restoration of bacterial sensitivity to colistin. The identification of rhein as a novel colistin potentiator offers a promising therapeutic strategy to address the growing challenge of colistin-resistant infections caused by *mcr-1* positive *E. coli*.

## Data Availability

The datasets presented in this study can be found in online repositories. The names of the repository/repositories and accession number(s) can be found in the article/Supplementary material.
