# Supplementary material for: Rhein restores the sensitivity of mcr-1 carrying multidrug-resistant Escherichia coli to colistin
Source: Front Microbiol. 2025 Jun 16;16:1586553. doi: 10.3389/fmicb.2025.1586553 (PMC12206717; doi:10.3389/fmicb.2025.1586553)
Supplement: Supplementary file 1 [file Table_1.docx]

Supplementary Material


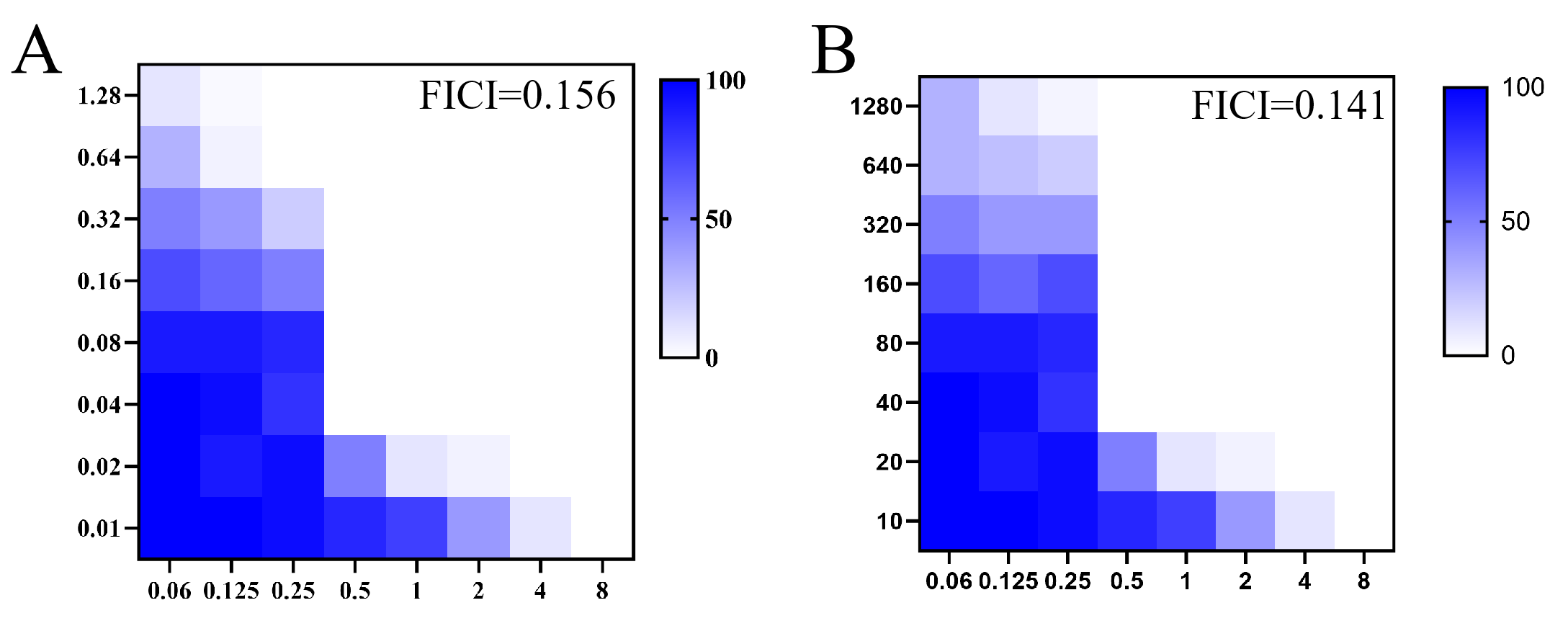


Figure S1 Growth inhibition checkerboards of rhein with colistin against *E. coli* B2 and 16QD. (A) *E. coli* B2 ; (B) *E. coli* 16QD.

**Supplementary Figure 1.** The figure legends are required to have the same font as the main text, 12 point normal Times New Roman, single spaced. Please use a single paragraph for each legend and prepare the figures keeping in mind the PDF layout.
